# Thermal and Mechanical Characterization of an Aeronautical Graded Epoxy Resin Loaded with Hybrid Nanoparticles

**DOI:** 10.3390/nano10071388

**Published:** 2020-07-16

**Authors:** Aldobenedetto Zotti, Simona Zuppolini, Anna Borriello, Mauro Zarrelli

**Affiliations:** Institute for Polymers, Composites and Biomaterials, National Research Council of Italy, 80055 Portici, Naples, Italy; aldobenedetto.zotti@unina.it (A.Z.); simona.zuppolini@cnr.it (S.Z.); mauro.zarrelli@cnr.it (M.Z.)

**Keywords:** polymer-matrix composites (PMCs), particle-reinforcement, fracture toughness, thermal properties, silica core/polydopamine shell nanoparticles

## Abstract

Synthesized silica nanoparticles (SiO_2_) were coated with a thin polydopamine (PDA) shell by a modified one-step procedure leading to PDA coated silica nanoparticles (SiO_2_@PDA). Core-shell (CSNPs) characterization revealed 15 nm thickness of PDA shell surrounding the SiO_2_ core (~270 nm in diameter). Different weight percentages of CSNPs were employed as filler to enhance the final properties of an aeronautical epoxy resin (RTM6) commonly used as matrix to manufacture structural composites. RTM6/SiO_2_@PDA nanocomposites were experimentally characterized in terms of thermal stability and mechanical performances to assess the induced effects by the synthesized CSNPs on pristine matrix. Thermal stability was investigated by thermogravimetry and data were modelled by the Doyle model and Kissinger methods. An overall enhancement in thermal stability was achieved and clearly highlighted by modelling results. Dynamic Mechanical Analysis has revealed an improvement in the nanocomposite performances compared to the neat matrix, with an increase in the glassy (+9.5%) and rubbery moduli (+32%) as well as glass transition temperature (+10 °C). Fracture Toughness tests confirmed the positive effect in damage resistance compared to unloaded resin with an impressive variation in critical stress intensity factor (K_IC_) and critical strain energy (G_IC_) of about 60% and 138%, respectively, with the highest SiO_2_@PDA content.

## 1. Introduction

The exceptional properties of composite materials compared to metals have allowed the former to be widely used as primary structures [1,2]. At the same time, several drawbacks of these materials prohibit their use in applications where their full potential could be exploited. For instance, in structural applications (transport or civil building) high standards of stability and durability are required. Furthermore, their low electrical and thermal conductivity, poor solvent resistance and vulnerability to fatigue damages strongly limit the use for these applications. Such drawbacks can be associated with the polymeric matrix, which is generally epoxy resin.

Although epoxy resins are characterized by a high level of processability (relatively low viscosity at low temperatures, curable even at room temperature), they are brittle in nature, due to the high level of crosslinking, and have low thermal stability [3]. Great efforts have been made to overcome the epoxy resins drawbacks without sacrificing their original performances: the most common approach consists of the addition of different typologies of fillers [4,5]. In the last few decades, many fillers have been employed as modifiers for epoxy resins, such as inorganic nanoparticles (NPs) [6], graphene nanoplatelets [7], rubber NPs [8], hyperbranched polymers [9] and CSNPs [5].

Filler parameters that critically influence the thermophysical and chemical performance of the corresponding nanocomposite are: dimensions (nanometric, sub-micrometric or micrometric); shape (spheres, whiskers or platelets); aspect ratio; dispersion in the matrix; chemical nature; surface functionality. The work of Morouf et al. [10] has demonstrated the effect of silica particles sizes on nanocomposites fracture energies. In fact, the reduction in the particle diameter (i.e., from 24 µm to 20 nm) allows us to obtain the same increase in fracture energy compared to the neat resin (i.e., 350%) using a filler weight content remarkably lower (i.e., from 65 wt% for micrometric NPs to 20 wt% for nanometric NPs). Kim et al. [11] have shown that the surface functionalization of carbon nanotube considerably increases the mechanical properties (in term of elastic modulus and tensile strength) of the hosting matrix, due to both the enhancement of filler-matrix adhesion and the improvement of filler dispersion.

The functionalization of particles is also commonly used during the synthesis of CSNPs. For example, Huang et al. [12] coated silica NPs with reduced graphene oxide (RGO), and the obtained CSNPs were employed as filler in an epoxy matrix. The RGO shell formation was possible thanks to the functionalization of silica surface with the (3-aminopropyl) triethoxysilane (APTES) coupling agent. Huang and coworkers also demonstrated that the formation of such core–shell nanostructures effectively prevents the aggregation of RGO nanosheets in a polymer matrix. A similar procedure has been employed by Zotti et al. [13], who synthetized CSNPs through the growth of an hyperbranched polymer on the functionalized surface of silica nanoparticles. These NPs were used as fillers in an aeronautical graded epoxy resin. The results have shown an improvement in fracture performances, attributable to the polymeric shell, and an increase in both thermal stability and storage modulus, attributable to the silica core nature.

It is well known [14] that an increase in filler–matrix adhesion can lead to an improvement in the mechanical performance of the corresponding nanocomposite. For this reason, in the last few decades researchers have extensively studied different techniques to improve filler–matrix adhesion (e.g., though NPs surface treatments with certain coupling agents (chemical bonding technique) [15] or using adhesive coating around NPs (physical integration technique) [16]). In the framework of adhesive coatings, Lee and coworkers [17] have reported a chemical procedure to synthesize functional coatings that mimic the adhesive proteins produced by mussels. Thanks to dopamine (DA) and other catechol compounds, mussels are capable of adhering to almost any surface, regardless of whether it is organic or inorganic. With the aim of imitating this mussel’s ability, many researchers have employed small molecules with catecholamine moiety for coating, obtaining NPs with improved adhesiveness properties. Moreover, these films can be easily deposited around the NPs’ cores though the oxidative self-polymerization of DA. Yang et al. [18] deposited a monolayer of PDA on clay surface, and the so modified clay was incorporated into an epoxy resin. It was found that the improved interfacial interactions between clay and matrix, due to the PDA layer presence, influenced not only the dispersion of the filler in the hosting matrix, but also the interfacial stress transfer, also inducing a remarkable improvement in thermomechanical properties at low filler weight content. Using a similar approach, Subramanian et al. [19] loaded an epoxy adhesive with PDA coated Multi Walled Carbon Nanotubes (MWCNT@PDA), in order to increase the lap shear strength of epoxy/aluminum joints. The results showed a doubled value of lap shear strength with only 0.5 wt% of MWCNT@PDA, and the authors explained this enhancement by an adhesive–cohesive force improvement mechanism.

In this work, an aeronautical graded epoxy resin was loaded with synthesized SiO_2_@PDA. A thin shell of PDA was employed in order to improve the interaction between filler and matrix. The effects of the synthesized CSNPs on thermal stability, mechanical behavior and fracture toughness performances of the hosting matrix were investigated considering the filler weight content as a variable. The results have been discussed and analyzed in comparison with data obtained by similar tests on neat epoxy matrix.

## 2. Materials and Methods

### 2.1. Materials

Tetraethyl orthosilicate (TEOS), APTES, ammonium hydroxide (NH_4_OH), dopamine (DA) and all solvents were purchased from Sigma-Aldrich (Milano, Italy). The used epoxy resin RTM6 was supplied by Hexcel Composites (Duxford, UK). RTM6 is a premixed epoxy-amine system, developed for the resin transfer molding process, and it is characterized by an epoxy equivalent weight of 116 g/eq [20] and a very low viscosity (33 mPa·s at 120 °C) [21].

### 2.2. SiO_2_@PDA Synthesis and Nanocomposites Manufacturing

**Figure 1:**
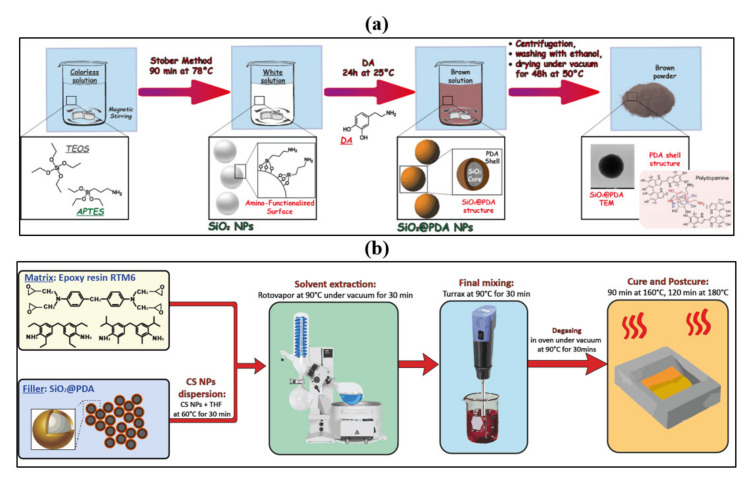
Scheme of (**a**) SiO_2_@PDA synthesis procedure and (**b**) SiO_2_@PDA/RTM6 nanocomposites manufacturing.

SiO_2_@PDA were synthesized by a one-step procedure optimized and characterized as reported in our previous work [22]. A scheme of the synthesis procedure is shown in Figure 1a. SiO_2_@PDA/RTM6 nanocomposites were manufactured as reported in our previous work [22] and are schematized in Figure 1b. A specific amount of CSNPs (i.e., 0.1, 1 and 5 wt% with respect to the epoxy resin).

### 2.3. SiO_2_@PDA Characterization

Fourier transform infrared (FTIR) analyses were performed using a Perkin Elmer Spectrum 100 FTIR spectrophotometer (Milano, Italy) within the range 4000–600 cm^−1^ (resolution 1 cm^−1^), using attenuated total internal reflectance spectroscopy (ATR). Bright field transmission electron microscopy (TEM) micrographs were obtained using a FEI Tecnai G12 Spirit Twin, equipped with a LaB6 source and a FEI Eagle 4K CCD camera (Eindhoven, The Netherlands).

### 2.4. Nanocomposites Thermal and Mechanical Characterization

Thermogravimetric analyses (TGA) were performed using a TGA Q500 system, by TA Instruments (New Castle, Germany). Tests were conducted under nitrogen flux (50 mL/min) using four different heating rates (5, 10, 15 and 20 °C/min). A sample weight of about 5 ± 0.5 mg was considered for each run test (from ambient temperature to 700 °C).

Dynamic mechanical analysis (DMA) was carried out using a TA Instruments Q800 DMA (New Castle, Germany) at standard frequency of 1 Hz, amplitude of 60 µm and a heating rate of 3 °C/min. The testing configuration was a double cantilever configuration with nominal sample dimensions of 60 × 10 × 2.5 mm^3^, according to ASTM D5023 standard. For each CSNPs concentration, three samples were tested for statistical purpose.

The fracture toughness performances of the manufactured nanocomposites were evaluated by using the Single Edge Notched Beam (SENB) specimen, according to the ASTM D5045-99 standard method. To ensure the plane strain conditions, samples with nominal dimensions of 3 × 6 × 27 mm^3^ were used. By following the indicated ASTM procedure, the crack length should be selected such that 0.45 < a/W < 0.55, where a is the crack length and W is the specimen width. The prescribed crack was obtained on each sample by a two-step procedure. Firstly, a sharp crack was machined by a controlled automatic sawing system, and then a natural crack was initiated using a razor blade across the crack tip. Fracture tests were performed by using a Lonos Tenso Test TT5 (Lonos Test, Monza, Italy) equipped with a 250 N load cell. The fracture toughness properties of a material are evaluated in terms of K_IC_ and G_IC_. The following equations were used to calculate K_IC_ values:

(1) $$K_{\mathrm{IC}}=f\left(\frac{a}{W}\right)\left(\frac{P_{Q}}{{\mathrm{BW}}^{1/2}}\right)$$

(2) $$f\left(\frac{a}{W}\right)=6{\left(\frac{a}{W}\right)}^{1/2}\frac{\left[1.99-\left(\frac{a}{W}\right)\left(1-\frac{a}{W}\right)\left(2.15-3.93\frac{a}{W}+2.7{\left(\frac{a}{W}\right)}^{2}\right)\right]}{\left(1+2\frac{a}{W}\right){\left(1-\frac{a}{W}\right)}^{3/2}}$$

where PQ is the load at failure and B is the specimen thickness. G_IC_ values were derived from the stress intensity values, according the following equation:

G_IC_ = U/BWΦ (3)

where U is the corrected energy and Φ is the energy calibration factor, both evaluated according the test standard. A displacement rate of 1 mm/min was employed and six separate measurements were performed to provide the average value and the error deviation for each CSNPs composition.

In order to study the fracture mechanisms involved during the nanocomposite failures, Scanning Electron Microscopy (SEM) observations were performed and analyzed. Micrographs of the nanocomposites fracture surfaces upon fracture tests, were carried out by FEI Quanta 200 FEG (ThermoFisher Scientific, Hillsboro, OR, USA).

## 3. Results and Discussion

### 3.1. SiO_2_@PDA Characterization

The SiO_2_@PDA were synthesized by a single step method [22]. After the nucleation and growth of the silica NPs, the DA monomer was directly added to self-polymerize into the oxidative environment reaction and the growth of the PDA shell occurred onto the silica core.

**Figure 2:**
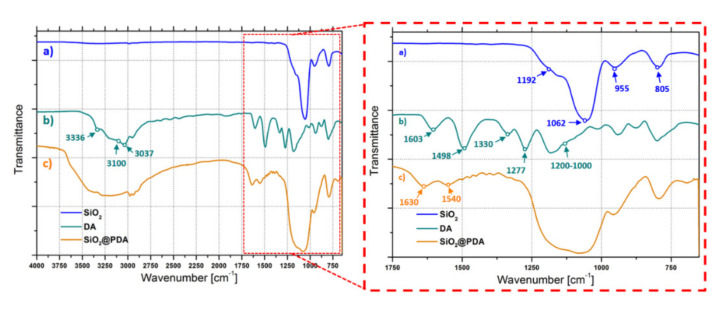
ATR–FTIR spectra of (**a**) pristine silica NPs, (**b**) DA and (**c**) SiO_2_@PDA.

The chemical structure of silica nanoparticles before and after PDA coating was investigated by ATR-FTIR measurements in the mid infrared region (4000–600 cm^−1^). Figure 2 illustrates the ATR-FTIR spectra of (a) pristine silica NPs, (b) DA and (c) SiO_2_@PDA. In the pristine silica spectrum, the high intensity band at 1062 cm^−1^, accompanied by a shoulder at 1192 cm^−1^, is attributable to the asymmetric stretching vibrations of the –Si–O–Si– groups. The characteristic peak of silanol groups is centered at 955 cm^−1^. The peak at lower wavenumber (805 cm^−1^) is associated to the asymmetric flexion of the –Si–O– bonds. At higher wavenumbers, the dopamine monomer spectrum is characterized by three main peaks centered at 3336 cm^−1^, 3100 cm^−1^ and 3037 cm^−1^, associated with the stretching vibrations of the –OH, –CN, and –NH groups, respectively. Minor peaks are observable in the 2750–2225 cm^−1^ range, corresponding to vibrations of either aryl or aliphatic C–H bonds. At lower wavenumbers, three main bands are observed at 1603 cm^−1^, 1498 cm^−1^ and 1277 cm^−1^, associated with the absorption peak of aromatic C=C, the twist vibration of N–H and the stretching vibration of the aryl oxygen, respectively. The peak at 1330 cm^−1^ is attributed to the hydroxyl groups’ vibrations. Peaks in the 1000–1200 cm^−1^ range are due to the in-plane bending vibrations of phenolic and –CH– groups. In the SiO_2_@PDA spectrum, the combined presence of both silica and DA peaks can be observed, suggesting the formation of the core/shell structure. The PDA formation is highlighted by the presence of indole and indoline aromatic ring vibration peaks centered at 1630 cm^−1^ and 1540 cm^−1^. The polymeric shell characteristic peaks show a lower relative intensity than those of the silica core, indicating the formation of SiO_2_@PDA with a small shell/core ratio.

**Figure 3:**
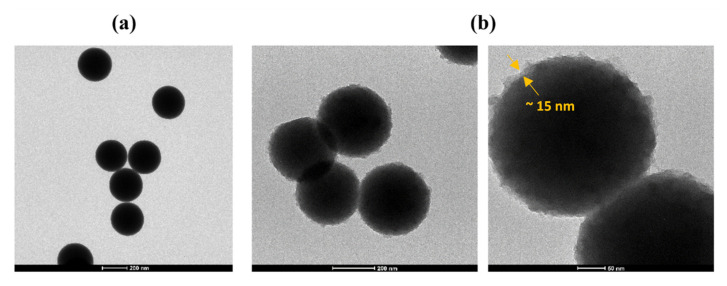
TEM micrographs of (**a**) bare silica NPs and (**b**) SiO_2_@PDA.

The ATR results are supported by TEM micrographs of the synthesized CSNPs. In Figure 3a, TEM images of silica NPs show particles with a very smooth surface and an average diameter of about 270 nm. The SiO_2_@PDA, as shown in Figure 3b, are characterized by an extremely rough surface and an increased average diameter (~300 nm), which confirms the presence of the PDA shell around the silica core. The PDA shell thickness is about 15 nm.

### 3.2. Thermogravimetric Analysis: Experimental Results and Kissinger Model

**Figure 4:**
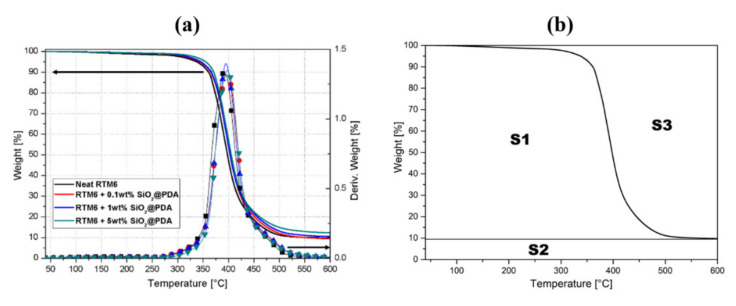
(**a**) TGA curves (nitrogen atmosphere) of neat epoxy and nanocomposites. (**b**) Schematic diagram of the Doyle’s method for determining the IPDT parameters.

The effect of SiO_2_@PDA addition on the thermal stability of the hosting matrix was investigated by TGA. Figure 4a shows the TGA thermograms of matrix and CSNPs loaded nanocomposites obtained at heating rate of 10 °C/min and under nitrogen atmosphere. All the analyzed systems show a considerable weight loss starting from 300 °C, associated with the degradation of the 3D macromolecular networks of the hosting matrix [23]. The maximum weight loss rate temperature, TMAX, the temperatures associated to a weight loss of 10% and 80%, T10 and T80 and the weight residual at 600 °C are summarized in Table 1. The characteristic degradation temperatures (TMAX, T10 and T80) of the nanocomposites underwent a small increase compared to the neat resin, highlighting the potential of the CSNPs to increase the thermal stability of the hosting matrix. Moreover, the minimum and maximum weight residual values are ~9% and ~13%, respectively, which are associated, respectively, with the neat resin and the sample RTM6 + 5 wt%SiO_2_@PDA. The difference in residual weight was an expected result considering the presence of an inert core (i.e., silica) within the SiO_2_@PDA.

The thermal stability effect of SiO_2_@PDA on RTM6 epoxy was also analyzed using an Integral Procedure Decomposition Temperature (IPDT) approach. IPDT, proposed by Doyle [24], is a reproducible parameter with practical meaning as an integrated half-volatilization temperature, and it is particularly useful for studying the thermal stability of polymers, regardless of the chemical nature and decomposition mechanism. Figure 4b reports a schematic diagram of Doyle’s method to evaluate the IPDT temperature, computed as follows:

IPDT = A* · K* · (T_f_ − T_i_) + T_i_ (4)

(5) $$A*=\frac{S_{1}+S_{2}}{S_{1}+S_{2}+S_{3}}\;K*=\frac{S_{1}+S_{2}}{S_{1}}$$

where A* is the ratio between the area subtended by the TGA curve and the total graph area, T_i_ is the initial temperature (40 °C), and T_f_ is the final temperature (600 °C). Table 1 reports the IPDT values for the neat matrix and the CSNPs loaded nanocomposites, respectively.

The IPDT temperature increases with the percentage of nanoparticle filler, revealing the higher thermal stability of the neat hosting matrix and this behavior is typical for systems loaded with inorganic fillers [25].

**Figure 5:**
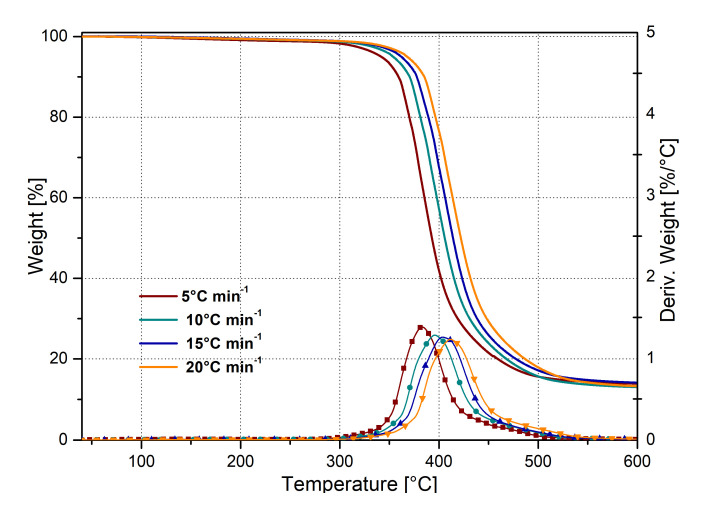
TGA curves of neat epoxy matrix at different heating rates.

To evaluate the activation energies without any pre-assumption on the thermal degradation mechanisms, the Kissinger Method (KM) was employed. Dynamic TGA experimental data were computed by using the KM model during dynamic decomposition. All tests were conducted at four different heating rates: 5, 10, 15 and 20 °C/min, respectively, as shown in Figure 5.

According to the KM model, the activation energy, E, can be evaluated by the linear plot of ln(β/T^2^_MAX_) vs. 1/T_MAX_ [26,27], as follows:

(6) $$\ln\left(\frac{\beta }{T_{\mathrm{MAX}}^{2}}\right)=\;\ln\frac{\mathrm{AR}}{E}-\frac{E}{{\mathrm{RT}}_{\mathrm{MAX}}}$$

where β is the constant heating rate employed during the TGA test, T_MAX_ is the maximum peak temperature (obtained by the derivative of the TGA curves), as shown in Figure 4 and Figure 5, and the ideal gas constant is identified by the symbol R.

**Figure 6:**
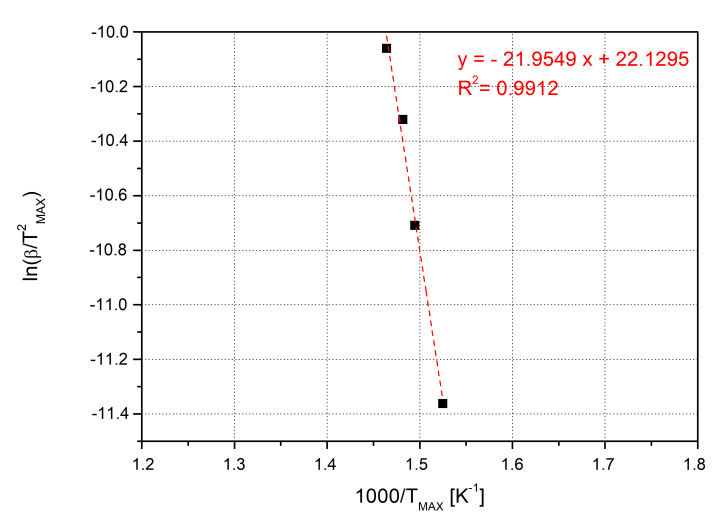
KM applied to the TGA experimental data of 5 wt% SiO_2_@PDA/RTM6 system.

Figure 6 reports the KM applied to the TGA experimental data in the case of 5 wt% of SiO_2_@PDA epoxy nanocomposites. The linear fitting is based on four computed points, one for each heating rate, and the R-squared (R^2^) value is equal to 0.9912.

**Figure 7:**
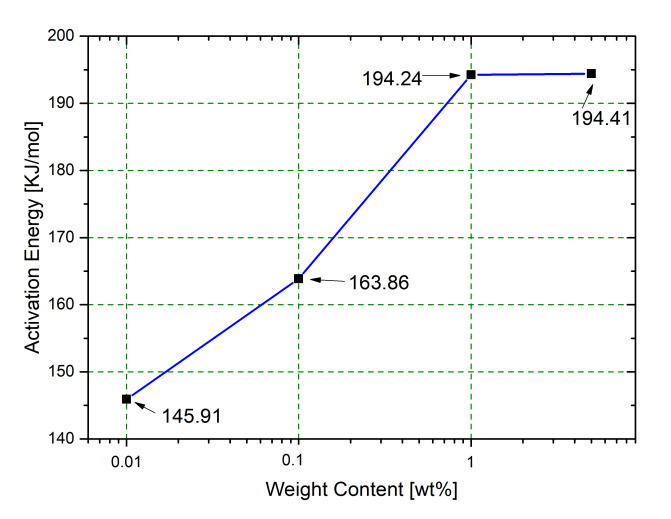
Degradation activation energies as function of nanocomposites filler content.

Figure 7 reports the Kissinger analysis results for neat RTM6 and SiO_2_@PDA/RTM6 systems. The activation energies of nanocomposites are higher compared to corresponding values for pure resin. The highest increase is associated to the system loaded with 5 wt% of SiO_2_@PDA, which is characterized by an activation energy equal to 194.41 KJ/mol. It is noteworthy that this value is similar to that of the sample loaded with 1 wt% NPs (i.e., 194.24 KJ/mol), indicating the achievement of a local maximum value for the degradation activation energy. This phenomenon could be caused by a worse dispersion of CSNPs in the sample loaded with highest filler content, as a coarser distribution of NPs in the hosting matrix can limit their efficiency as thermal stabilizer. The comparison of TGA results, IPDT parameters and degradation activation energies of the nanocomposites suggested that SiO_2_@PDA promote the thermal stability of final composite. This behavior can be attributed to both the core (silica) and the shell (PDA) of the employed filler. In fact, the incorporation of silica NPs plays a crucial role in improving the matrix thermal stability [28], as they act as effective physical barriers against the thermal decomposition of the hosting matrix. Moreover, it was demonstrated that PDA-based NPs/polymer nanocomposites are characterized by improved thermal stability performances compared to the neat systems [29], and this is likely due to the capability of PDA as melanin-like molecules [30] to eliminate (by radical addition or electron-transfer processes) the free radicals generated during the breaking of the C–C bonds.

### 3.3. Nanocomposites Dynamic-Mechanical Properties

The dynamic mechanical properties of the SiO_2_@PDA/RTM6 systems were examined within the temperature range 35–260 °C through DMA. The main results in terms of storage modulus at 35 °C (glassy modulus) and 260 °C (rubbery modulus), along with glass transition temperature (*T_g_*), are reported in Table 2.

**Figure 8:**
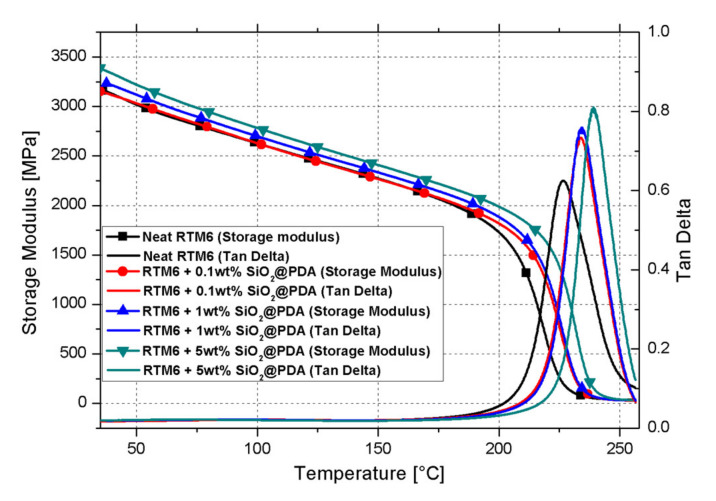
Dynamic mechanical analysis (DMA) curves of neat epoxy and SiO_2_@PDA/RTM6 systems.

Figure 8 shows the storage modulus and tan delta profiles as a function of temperature for neat epoxy and nanocomposites.

**Figure 9:**
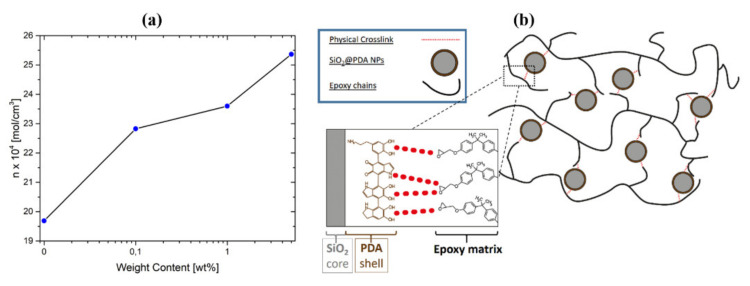
**(a)** Dynamic mechanical analysis (DMA) curves of neat epoxy and SiO_2_@PDA/RTM6 systems and (**b**) scheme of physical crosslinking of SiO_2_@PDA with epoxy chains.

Four main effects could be highlighted and discussed:

- Increase in glassy storage modulus: Different authors [31,32] have already reported this behavior in epoxy systems loaded with silica nanoparticles. The increase in storage modulus is attributable to the remarkable mismatch between the elastic modulus of epoxy matrix (3.19 GPa) and silica (70 GPa [33]);
- Increase in rubbery storage modulus: The reduction in crosslinking density in the hosting matrix can lower the elastic modulus in the rubbery region. Although the presence of the CSNPs actually limits the crosslinking mechanism of the epoxy polymeric chains, the CSNPs/RTM6 systems show an increase in the rubbery modulus with the increase in filler content. This behavior can be explained considering that the strong interfacial interactions, due to the PDA shell, allow the SiO_2_@PDA to act as a physical crosslinker. By a classical rubber elasticity equation [18] it is possible to estimate the apparent number of active network chain segments per unit volume (n):
  E = 3nRT(7)
  where E is the storage modulus at the onset of the rubbery plateau, R is the ideal gas constant and T is the absolute temperature corresponding to the E value. Figure 9a shows that n raises with the addition of CSNPs, demonstrating the ability of SiO_2_@PDA to act as a physical crosslinker, as shown in Figure 9b.
- Increase in T_g_: Different authors report that the addition of silica NPs can induce a reduction in *T_g_* [32] or it could keep it unchanged [34]. It is well known that the increase in *T_g_* in the case of a nanocomposite system likely depends by the ability of the filler to hinder the thermal motion of the polymeric hosting matrix chains. Thus, in the absence of bonds (physical or chemical) between hosting matrix and filler, the presence of the particles can induce a detrimental effect on the glass transition temperature of the neat matrix. Since SiO_2_@PDA acts as a physical crosslinker, the motion of the polymeric chains is efficiently hindered by their presence, with a consequent increase in the final glass transition temperature of the nanocomposite system;
- Increase in tan delta peak height: Tan delta is expressed as the ratio between the dissipative modulus (E’’) and the storage modulus (E’), and consequently the height of the tan delta peak is a measure of the material damping performance. Allahverdi et al. [35] have demonstrated that the addition of silica NPs will induce a reduction in the tan delta peak height, and this behavior is attributed to the inorganic nature of the silica particles which prevents energy dissipation. The presence of the PDA shell, instead, ensures an efficient dissipation due to its polymeric (and consequently viscoelastic behavior) nature, confirming the clear enhancement of damping performance for the SiO_2_@PDA/RTM6 nanocomposites.

### 3.4. Nanocomposites Fracture Toughness Performances

Fracture toughness tests were performed by using SENB specimens in a 3-point bending configuration, according to the ASTM D5045 test standard. The cross-head displacement rate employed is 10 mm·min^−1^. During the 3-point bending tests for the determination of fracture toughness properties, all samples (neat epoxy and nanocomposites) exhibited a typical linear load-displacement behavior up to failure. The maximum loads were used to evaluate the fracture toughness together with the initial crack length and the sample dimensions. Two main parameters describe the fracture toughness performances of a material—K_IC_, which represents the critical stress amplification factor near the edge of a crack, and G_IC_, which is the energy for creating two surfaces during the fracture propagation.

**Figure 10:**
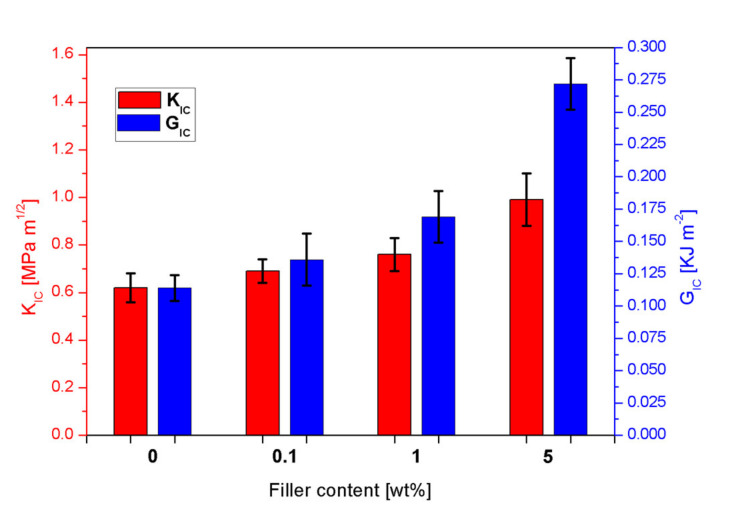
K_IC_ and G_IC_ values obtained from fracture toughness tests for neat epoxy and nanocomposites.

Figure 10 shows both K_IC_ and G_IC_ values for the neat matrix and the SiO_2_@PDA/RTM6 systems. The addition of CSNPs remarkably increases both the parameters, and the highest improvement is achieved for the samples loaded with the highest filler content (i.e., 5 wt%). Table 3 reports the fracture toughness test results. It is observed that, with 5 wt% of CSNPs, the value of K_IC_ is improved from 0.62 MPa·m^1/2^ to 0.99 MPa·m^1/2^, which corresponds to an increase of 60%.

Concerning the G_IC_ values, the highest increase compared to the neat matrix is equal to 139%, associated with the sample loaded with 5 wt% of SiO_2_@PDA. The outstanding improvement in fracture toughness performances is attributable to different mechanisms:

- Crack pinning is a typical fracture mechanism in polymeric systems loaded with inorganic particles. Hard fillers obstruct the propagation of the crack front, increasing the fracture toughness by bowing out the crack front between the NPs [36];
- The strong adhesion between filler and matrix promotes a severe triaxial stress state around the CSNPs, inducing a plastic deformation mechanism in the hosting matrix and causing crack tip blunting [37];
- The further plastic deformation of the matrix due to the presence of the PDA shell itself [38].

**Figure 11:**
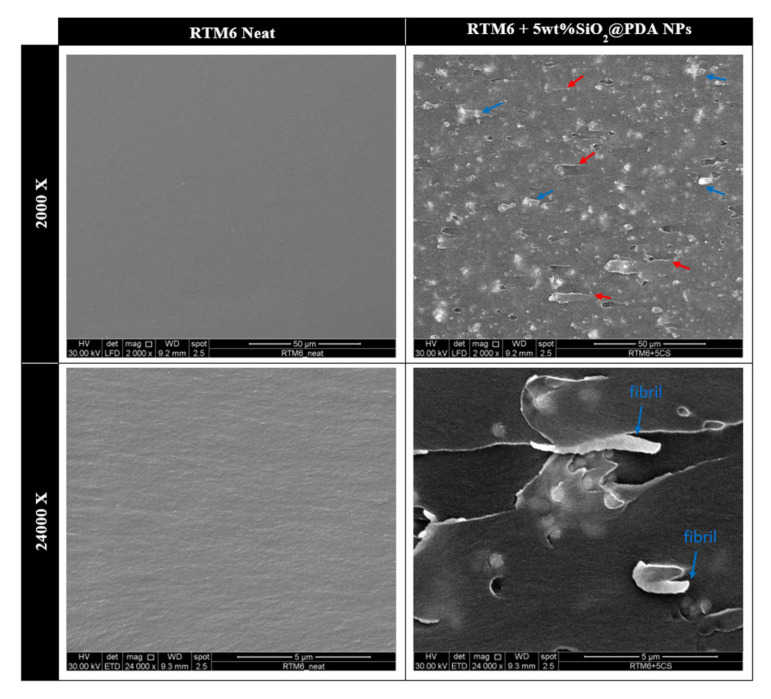
SEM images of neat epoxy and 5 wt% SiO_2_@PDA loaded system fracture surfaces labeled according to row and column indication.

In order to assess the micro-mechanical mechanisms of failure, the fracture surfaces of tested samples were analyzed using SEM, as shown in Figure 11. The neat epoxy fracture surface appears extremely smooth, indicating the brittle nature of the epoxy matrix. The addition of 5 wt% SiO_2_@PDA remarkably increases the roughness of the fracture surfaces and, in particular, two main features can be highlighted—crack pinning tails (red arrows), associated with the crack pinning mechanism, and plastic deformation areas/fibrils (blue arrows), attributable to the plastic deformation mechanism. These characteristics are in agreement with the previously purposed fracture mechanisms, supporting the large increase in critical released energy during the formation of new surfaces compared to the neat matrix.

The presence of agglomerates of SiO_2_@PDA observable in the 5 wt% SiO_2_@PDA loaded system can be explained considering that the large number of catechol and amine groups on the CSNPs surface not only promotes their dispersion in the hosting matrix but also their adhesion between adjacent NPs. Thus, the self-agglomeration of SiO_2_@PDA occurs with the formation of small clusters in the epoxy nanocomposites.

## 4. Conclusions

The effects of synthesized silica core/polydopamine shell NPs on thermal, mechanical and fracture properties of an aeronautical graded epoxy resin have been investigated. SiO_2_@PDA synthesis was performed by a single step procedure. Thermal stability was studied by TGA, and the results have shown that the addition of 5 wt% of CSNPs induces a slight improvement of both IPDT and degradation activation energy compared to the neat matrix.

Dynamical mechanical properties were evaluated using DMA, and tests conducted in a 3-point bending configuration have revealed a global improvement with the addition of the highest filler content, in terms of the moduli, *T_g_* and height of Tan delta peak. In particular, the storage modulus at 30 °C increases by about 8% and the *T_g_* of the nanocomposite is 10 °C higher than the neat matrix. Fracture toughness results indicate an improvement in the material performances with an increase in both K_IC_ and G_IC_ of 60% and 139%, respectively. Crack pinning and plastic deformation have been identified as the mechanisms causing the fracture toughness performances improvement, and these hypotheses were verified through SEM observations.

## Figures and Tables

**Table 1:** Results of TGA tests (in nitrogen) for neat epoxy and SiO_2_@PDA/RTM6.

Sample	T^10^ (°C)	T^80^ (°C)	T_MAX_ (°C)	IPDT (°C)	Weight Residual (@ 600 °C) (%)
Neat RTM6	360.5 ± 1.3	444.4 ± 1.0	391.8 ± 0.1	479.7	9.05 ± 0.3
RTM6 + 0.1 wt%SiO_2_@PDA	363.4 ± 1.2	454.4 ± 5.5	394.5 ± 0.9	482.7	9.09 ± 0.2
RTM6 + 1 wt%SiO_2_@PDA	365.0 ± 0.9	455.9 ± 0.8	394.6 ± 0.9	492.8	10.19 ± 0.5
RTM6 + 5 wt%SiO_2_@PDA	370.5 ± 0.5	464.7 ± 2.6	395.9 ± 1.1	510.5	13.03 ± 0.3

**Table 2:** Results of DMA tests for neat epoxy and SiO_2_@PDA/RTM6 systems.

Sample	Storage Modulus (at 30 °C) (MPa)	Storage Modulus (at 260 °C) (MPa)	*T_g_* (°C)
Neat RTM6	3192 ± 21.0	25.1 ± 1.5	228.9 ± 0.5
RTM6 + 0.1 wt% SiO_2_@PDA	3200 ± 16.0	29.5 ± 1.5	233.6 ± 0.2
RTM6 + 1 wt% SiO_2_@PDA	3275 ± 19.0	30.5 ± 1.0	234.3 ± 0.4
RTM6 + 5 wt% SiO_2_@PDA	3434 ± 8.0	33.1 ± 0.7	239.0 ± 0.2

**Table 3:** Results of fracture toughness tests for neat epoxy and SiO_2_@PDA/RTM6 systems.

Sample	K_IC_ (MPa·m^1/2^)	% Variation K_IC_	G_IC_ (KJ·m^2^)	% Variation G_IC_
Neat RTM6	0.62 ± 0.06	-	0.114 ± 0.01	-
RTM6 + 0.1 wt%SiO_2_@PDA	0.69 ± 0.05	11.3	0.136 ± 0.02	19.3
RTM6 + 1 wt%SiO_2_@PDA	0.76 ± 0.07	22.6	0.169 ± 0.02	48.3
RTM6 + 5 wt%SiO_2_@PDA	0.99 ± 0.11	60.0	0.272 ± 0.02	138.6
